# Ferroelectric Domains and Evolution Dynamics in Twisted CuInP_2_S_6_ Bilayers

**DOI:** 10.1002/smtd.202500683

**Published:** 2025-06-20

**Authors:** Dongyu Bai, Junxian Liu, Yihan Nie, Yuantong Gu, Dongchen Qi, Arkady V. Krasheninnikov, Liangzhi Kou

**Affiliations:** ^1^ School of Mechanical Medical and Process Engineering Queensland University of Technology Brisbane Queensland 4001 Australia; ^2^ College of Civil Engineering and Architecture Zhejiang University Hangzhou 310058 China; ^3^ School of Chemistry and Physics Queensland University of Technology Brisbane Queensland 4001 Australia; ^4^ Institute of Ion Beam Physics and Materials Research Helmholtz‐Zentrum Dresden‐Rossendorf 01328 Dresden Germany

**Keywords:** deep learning, domain dynamics, interfacial coupling, polar domains, twisted bilayer

## Abstract

Polar domains and their manipulation—particularly the creation and dynamic control—have garnered significant attention, owing to their rich physics and promising applications in digital memory devices. In this work, using density functional theory (DFT) and deep learning molecular dynamics (DLMD) simulations, it is demonstrated that polar domains can be created and manipulated in twisted bilayers of ferroelectric CuInP_2_S_6_, as a result of interfacial ferroelectric (antiferroelectric) coupling in AA (AB) stacked region. Unlike the topological polar vortex and skyrmions observed in superlattices of (PbTiO_3_)_n_/(SrTiO_3_)_n_ and sliding bilayers of BN and MoS_2_, the underlying mechanism of polar domain formation in this system arises from stacking‐dependent energy barriers for ferroelectric switching and variations in switching speeds under thermal perturbations. Notably, the thermal stability and polarization lifetimes are highly sensitive to twist angles and temperature, and can be further manipulated by external electric fields and strain. Through multi‐scale simulations, this study provides a novel approach to exploring how twist angles influence domain evolution and underscores the potential for controlling local polarization in ferroelectric materials via rotational manipulation.

## Graphical abstract

A novel mechanism to control ferroelectric and antiferroelectric domains in bilayer twisted CIPS via Moiré superlattices and interlayer coupling. Combined DFT and MLMD simulations reveal the twist‐angle‐dependent domain evolution, offering mechanistic insights and design principles for controllable domain switching and programmable nano electronic devices based on low‐dimensional materials.

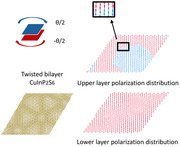

## 1 Introduction

Polar domains or vortices, referring to regions with uniformly aligned electric dipoles, represent a vibrant research frontier in condensed matter physics, as evident from the discoveries of domain‐induced negative capacitance,^[^ ^1^, ^2^, ^3^, ^4^ ^]^ topological skyrmionic states,^[^ ^5^, ^6^, ^7^, ^8^ ^]^ and unconventional phase transitions.^[^ ^9^, ^10^, ^11^, ^12^ ^]^ Understanding the microscopic and macroscopic characteristics of these topological phenomena, precisely manipulating these domains, as well as revealing critical factors influencing their stability and morphology, are of fundamental and practical interest.^[^ ^13^ ^]^ Traditionally, stress–strain effects have been employed to facilitate the formation of polar topologies and the annihilation of the polarization domain. For example, lattice mismatch strain in heterostructures (PbTiO_3_)_n_/(SrTiO_3_)_n_ has enabled the realization of stable polarization vortices or polar skyrmions within ferroelectric bubble domains at room temperature. ^[^ ^5^, ^14^ ^]^ This behavior is driven by the competition between electrostatic and strain boundary conditions, which inhibits the formation of a homogeneous ferroelectric ground state. The strain engineering techniques are recently also utilized to induce localized spontaneous polarization switching in van der Waals (vdW) ferroelectric materials, such as In_2_Se_3_ and CuInP_2_S_6_ (CIPS). Localized bending and bubbling under high‐strain conditions lead to the formation of stable polarization domains.^[^ ^15^, ^16^, ^17^ ^]^ However, it is noticed that strain engineering has its inherent limitations. The ability to precisely tune strain gradients via mechanical boundary conditions is often restricted, complicating control over polarization switching.^[^ ^18^, ^19^ ^]^ Furthermore, in heterostructures, imperfections at the surfaces and interfaces significantly affect interface coupling,^[^ ^20^ ^]^ with defects and dislocations impeding domain nucleation or influencing domain wall motion, thereby reducing overall domain stability.^[^ ^21^ ^,^ ^22^ ^]^

Associated with the discovery of superconductivity in twisted bilayer graphene, twist deformation has recently emerged as an effective approach to regulate interlayer coupling in low‐dimensional ferroelectric materials.^[^ ^23^, ^24^, ^25^ ^]^ The induced Moiré superlattices can enhance interlayer quantum coupling and electron interactions to form stable polar topologies. For example, tunable topological vortices have been observed in twisted bilayer MoS_2_ structures,^[^ ^26^, ^27^ ^]^ while rotational polarization textures are generated in twisted freestanding BaTiO_3_ layers due to nanoscale strain modulation.^[^ ^19^ ^]^ Additionally, alternating polar domains with anti‐aligned dipoles have been identified in twisted WSe_2_.^[^ ^28^, ^29^ ^]^ These discoveries highlight the potential of twist‐induced Moiré superlattices for the electrical control of ferroelectric domains. Nevertheless, experimental challenges persist, particularly in achieving high‐stable ferroelectric domain and understanding the atomic‐level coupling between twist angles and polarization switching.^[^ ^30^, ^31^, ^32^ ^]^

Here, we predict the existence of stable polar domains in bilayer twisted CIPS and investigate their domain evolution dynamics under external driving forces through joint density functional theory (DFT) and molecular dynamics (MD) simulations. Static DFT calculations reveal that the stacking order in bilayer CIPS modifies the interlayer electron distribution, resulting in a lower polarization switching barrier for the ferroelectric (FE) to antiferroelectric (AFE) transition in the AB stacking compared with AA stacking. As a result, FE/AFE domains are localized around AA/AB stacking regions in twisted CIPS bilayers, as seen from large‐scale MD simulations based on the developed deep learning potential.^[^ ^33^, ^34^, ^35^ ^]^ The thermal stability of the FE domain and polarization lifetimes are highly sensitive to twist angles and temperature. They can be further enhanced or weakened through external electric fields and strain. These results not only deepen the understanding of FE domain formation and manipulation in low‐dimensional systems but also highlight the potential of 2D ferroelectric materials in next‐generation electronic devices, particularly in data storage and sensor technologies.

## 2 Results and Discussion

### 2.1 Switching Barrier Influenced by Stacking

**Figure 1:**
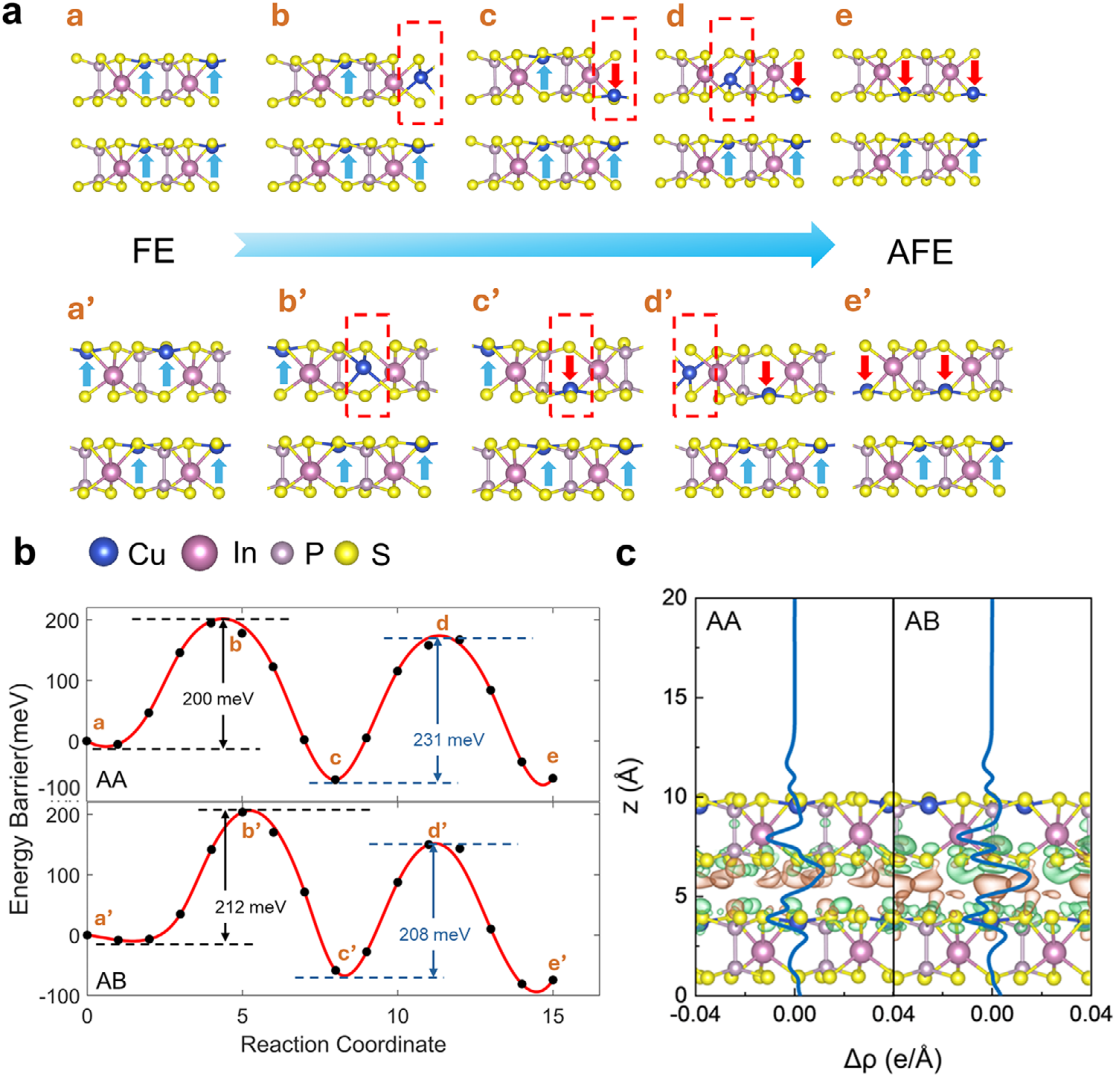
DFT results for different stacking orders in bilayer CIPS. a) The minimum energy paths for Cu displacement in AA & AB stacked 2 × 1 bilayers. b) The energy barriers for FE ↔AFE transitions in AA & AB stacked bilayers. c) Planar‐averaged electron density difference Δρ(z) for CIPS as a function of the z coordinate which is perpendicular to the atomic planes. The green and orange areas indicate electron depletion and accumulation, respectively.

2D CIPS is a vdW ferroelectric material with experimentally confirmed room‐temperature stability.^[^ ^36^ ^]^ The spontaneous polarization originates from the broken structural symmetry along the out‐of‐plane direction, it is in a FE phase when Cu ions are located on the top surface. The polarization reversal can be achieved with the vertical displacement of the Cu ions within the octahedron.^[^ ^37^ ^]^ The large energy barrier (≈200 meV) associated with Cu migration ensures the stability of the FE state at room temperature.^[^ ^38^ ^]^ When one CIPS layer is stacked on top of another, the resulting bilayer may exhibit either a FE or AFE phase, depending on the relative positions of the Cu cations in the two layers. In the AFE phase, the Cu cations in the upper and lower layers are aligned in a head‐to‐head configuration (**Figure** **1**a, structure e, e’), with the polarizations of the two layers pointing in opposite directions. In contrast, the Cu cations adopt a head‐to‐foot configuration in the FE phase, resulting in an enhanced overall polarization (Figure 1a, structure a, a’).

A phase transition between FE and AFE states can be achieved when the Cu cation in one layer moves from the top to the bottom site within the octahedron. Within a 2 × 1 supercell, the polarization reversal exhibits a bimodal behavior with two energy barriers due to the presence of metastable structure c/c' (Figure 1a). More interesting, the energy barriers are stacking‐dependent, as shown in Figure 1b. Under AA‐stacking configuration (each atom in the upper layer is directly aligned with the corresponding atom in the lower layer), the energy barriers for a Cu ion motion within the upper CIPS layer are 200 meV (a → c transition) and 231 meV (c → e transition), respectively. As a comparison, the values in the AB‐stacked configuration (Cu/In atoms in the upper layer are located directly above the S atoms in the lower layer) are 212 and 208 meV, respectively. This implies that, although the initiation of Cu migration for polarization reversal in AB stacking is relatively more difficult compared with AA configuration (200 vs 212 meV), the following displacement and associated polarization reversal will be much easier (231 vs 208 meV). The difference originates from the stacking‐dependent interfacial interactions and electron distributions. DFT calculations with vdW corrections indicate that the interlayer distance for AA/AB stacking is 3.24/3.18 Å. As a result, compared with AA stacking, more holes in AB structure are accumulated at the interlayer interface and more electrons gather between the layers (Figure 1b). This net charge buildup creates an internal electric field between the layers, which facilitates the movement of Cu cations, leading to an overall lower polarization switching barrier for AB stacking. The stacking‐dependent switching barrier in bilayer CIPS implies the potential to induce FE domain and coexisted FE/AFE phase when it undergoes interfacial twist to create the Morié pattern.

Besides, the distribution of polarization domains is influenced by the relative energetic stability of different stacking configurations. As shown in Figure S1 (Supporting Information), for the AA stacking, the energy is −110.385 eV in the FE state and −110.447 eV in the AFE state (per unit cell). In comparison, the AB stacking exhibits consistently lower total energies in both cases (−110.442 eV for FE and −110.516 eV for AFE). The energy difference between AA and AB stackings is ≈70 meV per unit cell in the AFE state, and 56 meV per unit cell in the FE state. This indicates that the CIPS bilayer thermodynamically favors the lower‐energy AB‐stacked regions. This also supports the conclusion that AFE domains preferentially form and stabilize in AB regions.

### 2.2 Deep‐Potential Predicted Switching Properties

Static DFT simulations can accurately describe the energetics and electronic structure of the system, however, their inherent limitations related to high computational cost prevent them from mimicking complex Moiré superlattice systems and studying thermal disturbance and dynamic process which more effectively reflect the real‐world impacts. Here, the force fields of CIPS are developed using the DeepMD‐kit^[^ ^33^ ^]^ to enable large‐scale MD simulations, with a training dataset derived from the open‐source data^[^ ^39^ ^]^ as well as our DFT calculations. Energies, forces, and atomic coordinates for each high‐symmetry stacking configuration are calculated using vdW‐corrected DFT approach to capture the delicate interlayer interactions. These values are then used as inputs for the machine learning model, ensuring the accuracy of subsequent DeepMD simulations in reproducing the stacking‐dependent interlayer interactions. The stacking order within local regions of the Moiré pattern was determined by laterally shifting the top layer, as illustrated in Figure S2 (Supporting Information). Our benchmark calculations indicate that the developed force field performs remarkably well in both energy and force predictions, with the mean absolute error (MAE) in energy of ≈1.26 meV atom^−1^ (Figure S3, Supporting Information).

**Figure 2:**
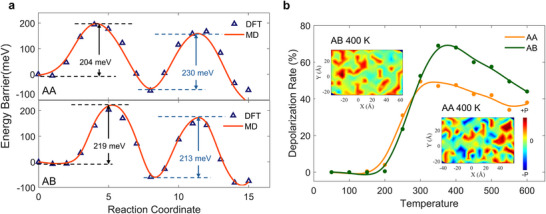
DLMD results for different stacking orders in bilayer CIPS. a) The switching barrier for FE ↔ AFE phase transition in AA and AB structures, as reproduced from DLMD simulations. The triangles represent the NEB results from the DFT calculations, while the line indicates the barrier calculation curve from the DLMD potential. b) Depolarization rate of AA and AB stacked structures as a function of temperature at 100 ps. The insets show the polarization distribution diagrams of both structures at 400 K.

The developed Deep Potential (DP) force field demonstrates exceptional accuracy in predicting both energies and forces (Figures S3 and S4, Supporting Information). It can predict the energies of the metastable AA and ground‐state AB stacking configurations with remarkable precision, yielding errors of only 0.006% and 0.02%, respectively, relative to DFT values (Figure S5, Supporting Information). The transition process of Cu cation movement is also accurately reproduced by the DP force field, with energy profiles from both methods being nearly identical. Specifically, the switching barriers are 204 and 230 meV for the AA‐stacked structure, 219 and 213 meV for the AB‐stacked structure respectively. This perfect agreement highlights the high accuracy and reliability of the developed potential and its capability to model the dynamic evolution of phase transitions from the FE state to the AFE state under thermal disturbances (**Figure** **2**a).

Given the lower energy barrier, ferroelectric polarization in the AB stacking configuration is expected to depolarize more easily under external thermal disturbance. To test this hypothesis, we conducted DeepMD simulations using 10 × 10 × 1 supercells, initializing the two layers with a head‐to‐foot polarized configuration to study the domain evolution dynamics in both stacking structures (Figure 2b). The results indicate that the polarizations of both configurations remain stable and unreversed until the temperature increases to 150 K, suggesting that thermal disturbances at this level are insufficient to overcome the energy barrier for ferroelectric switching. As the temperature rises further, the depolarization rate, defined as the average percentage of AFE domain formation (depolarization) per picosecond, diverges between the two configurations. Due to its lower resistance to thermal perturbations (the first energy barrier is relatively smaller), AA‐stacked structure exhibits a slightly faster depolarization rate than AB stacking within the temperature range of 150–270 K, facilitated by the increased likelihood of intermediate‐state formation. Under higher temperature (above 270 K), the AB‐stacked structure, benefiting from a lower secondary energy barrier, undergoes a significant increase in depolarization rate, leading to the formation of larger AFE domains, as shown in the polarization distribution at 400 K and 100 ps. In contrast, the AA‐stacked structure exhibits limited AFE domain expansion, with extensive polarization retention and reversal events. These results validate the static and dynamic stacking‐dependent polarization switching behaviors observed in both DFT and DeepMD simulations, further implying the potential to create and manipulate polarization domains in complex systems, such as twisted bilayer structures.

### 2.3 Twist‐Induced Polarization Domain and Thermal Stability

**Figure 3:**
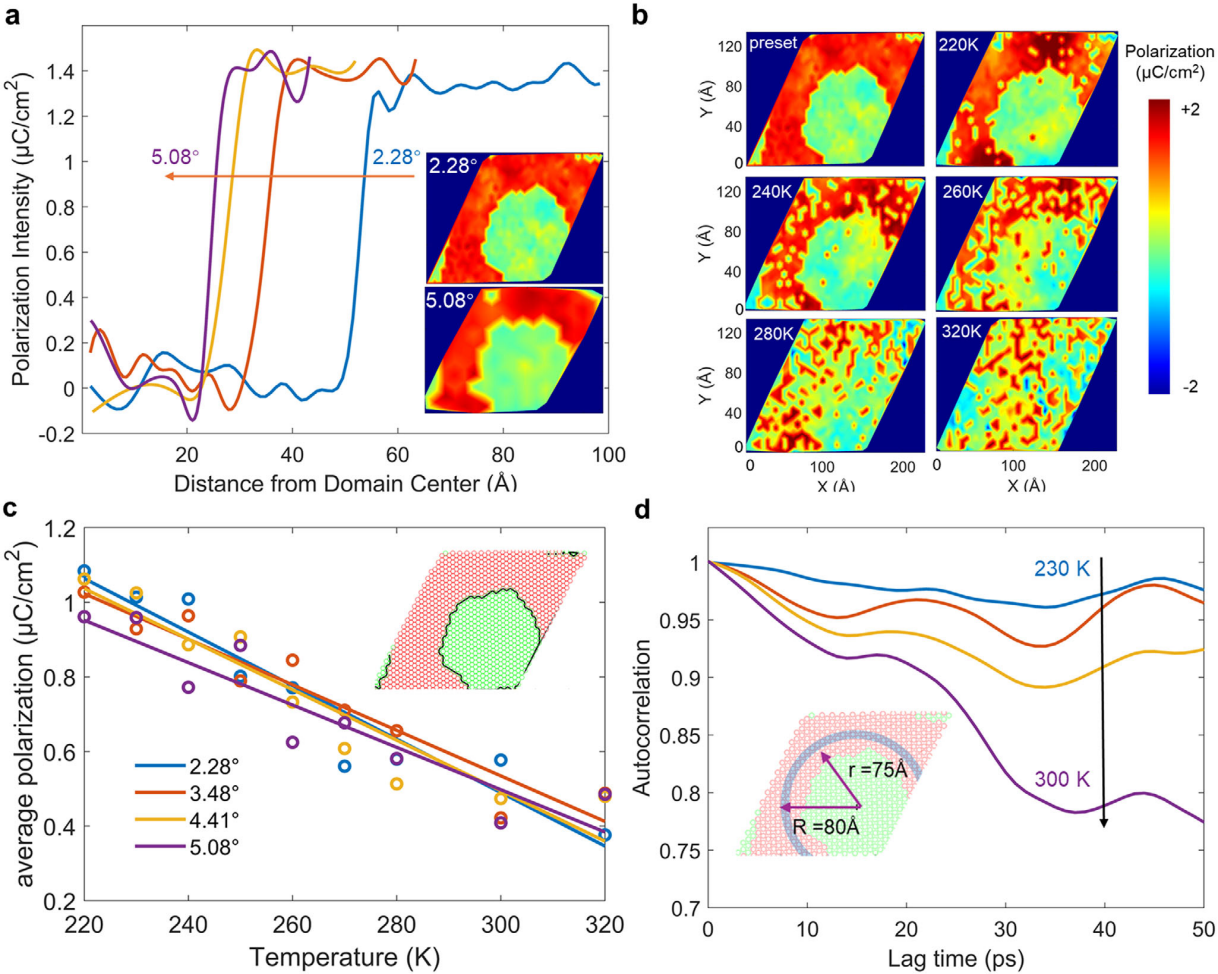
FE domain and dynamics in Moiré twist. a) Polarization distribution for twisted bilayers under static conditions. b) Heatmap of the polarization distribution at various temperatures for a twisted bilayer with a twist angle of 2.28°. c) Average polarization as a function of temperature. The illustration highlights the boundary of the polarization domain at 0 K, with the red region representing the initial FE domain. d) Temperature‐dependent correlation function of polarization, with the polarization averaged over the blue region.

Here, a twisted CIPS bilayer structure is built to explore the influence of stacking on polarization domain formation, manipulation, and the thermal stability of these domains. Four models with twist angles of 2.28°, 3.48°, 4.41°, and 5.08° (Figure S6, Supporting Information) were constructed for large‐scale DeepMD simulations. The resulting Moiré superlattice exhibits a coexistence of AA and AB stackings, with a distinct boundary separating the regions. After full relaxation, atomic reconstruction occurs, characterized by the expansion of low‐energy AB domains and the corresponding shrinkage of high‐energy AA domains. (Figure S7, Supporting Information) Based on recent experimental findings (the interlayer is in FE coupling in bulk or multiple layer CIPS^[^ ^36^ ^]^) and stacking‐dependent stability as above, compared to AA stacking region AB will be more likely to be switched into AFE coupling as temperature increases. Thus, the stacking‐dependent interlayer coupling induces polarization domains in the AA‐stacked region, but non‐polarized (AFE coupling) in the AB‐stacked region (Figure S8, Supporting Information). The FE domains in the AA region depend on the twist angle, where a decrease in the twist angle leads to an increase in the AFE domain size. The average radius of non‐polarized domains decreases from 53 to 22 Å as the twist angle increases from 2.28° to 5.08° (**Figure** **3**a).

DeepMD simulations were conducted on twisted models at external temperatures to investigate the thermal stability of polar domains. Snapshots of polarization domain evolution at 100 ps for the 2.28° twisted bilayer under various temperatures are shown in Figure 3b and Figures S9–S12 (Supporting Information). At 220 K, while thermal perturbations increase the atomic energy, it remains insufficient to overcome the energy barrier required for polarization reversal in the AA‐ and AB‐stacked configurations. As a result, both FE and AFE domains maintain relatively stable. At 260 K, however, polarized domains undergo significant disruption, part of AA configurations are transited from the FE to AFE state, as thermal fluctuations destabilize the FE domain. As temperature further increases, both AFE and FE domains are destroyed in the twisted structure, and no clear domain boundary can be observed (at 320 K). The average polarization as a function of temperature was calculated across different twist angles (Figure 3c) to quantify the impacts of thermal disturbance on the domain stability. It decreases with increasing temperature in a nearly linear trend, indicating a uniform thermodynamic response of AA‐stacked structures with the same twist angle to thermal fluctuations below 320 K. Notably, polarization regions associated with smaller twist angles exhibit higher thermodynamic stability, whereas configurations with larger twist angles are more prone to disruptions from thermal fluctuations (Figure 3c).

Its lifetime is examined by the autocorrelation of the polarization distribution function within the domain relative to the initial states. As shown in Figure 3d, the autocorrelation remains nearly 100% at 230 K after 50 ps, indicating the polarization state keeping unchanged for 50 ps, namely its high stability over time. However, obvious polarization reversal can be observed within the domain at 300 K, as evidenced by a significant decrease in autocorrelation, ≈77% dropping at 50 ps. This reduction in polarization lifetime at higher temperatures reflects the difficulty in maintaining stable domain walls and nuclei, as seen in the disrupted FE and AFE domains at 320 K (Figure 3b). As such, our findings demonstrate that both temperature and twist angle play a crucial role in determining the stability of polarized domains.

### 2.4 Modulation of Polarization Domain by External Fields

**Figure 4:**
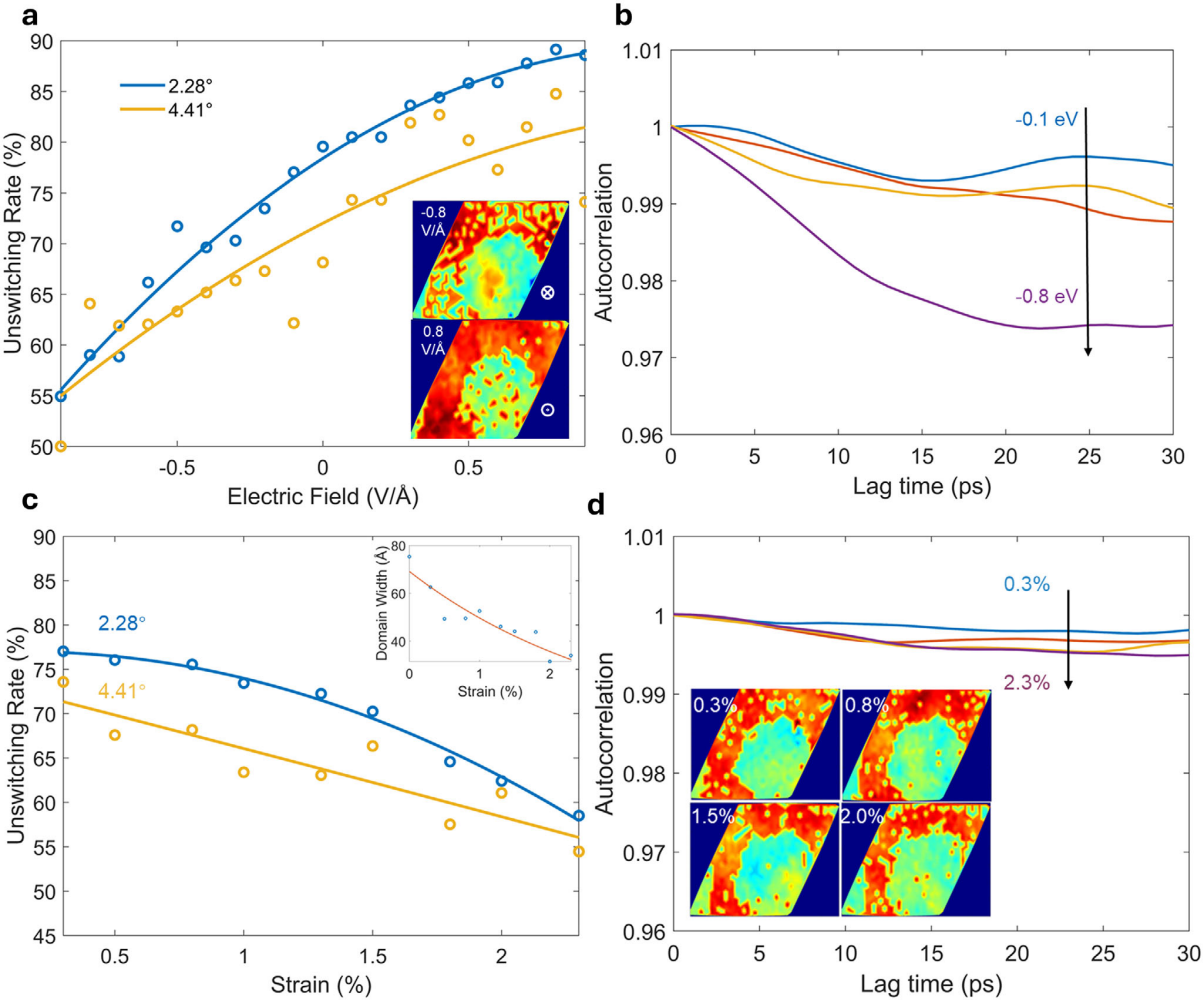
Polarization domain behavior in Moiré twist under external field. a) Percentage of structures retaining polarization unchanged in a twisted Moiré bilayer CIPS as a function of a perpendicular electric field at 220 K. Insets show heatmaps of the polarization distribution under electric fields of ±0.8 V Å^−1^ for a structure with a twist angle of 2.28°. b) Correlation function of polarization as a function of electric field strength. c) Percentage of structures retaining polarization in a twisted Moiré bilayer CIPS as a function of strain at 220 K. Inset depicts changes in FE domain width under strain for the 2.28° model. d) Strain‐dependent correlation function of polarization. The illustrations include heatmaps of the polarization distribution at strain levels of 0.3%, 0.8%, 1.5%, and 2.0%.

External electric field is commonly used to manipulate FE domains, as demonstrated in twisted MoS_2,_ WSe_2_, and BN.^[^ ^27^, ^29^, ^40^ ^]^ Here the manipulation of electric field on the polarization domain, focusing on stability, lifetime, and size is studied in twisted CIPS bilayers. DFT simulations (Figure S13, Supporting Information) revealed that the first energy barriers for FE‐AFE phase transitions in both AA‐ and AB‐stacked bilayers linearly increase or decrease under positive or negative electric fields, respectively. This behavior results from the fact that a positive/negative electric field suppresses/facilitates Cu ion migration due to Coulombic interactions. It also implies the effective manipulation of the electric field on FE domain in twisted bilayer. Here, polarization domain evolution in twisted bilayers (2.28° and 4.41°) was studied from DeepMD simulations at 220 K under vertical electric fields ranging from ±0.1 to ±0.9 V Å^−1^. The effect of the electric field was modelled as an equivalent force exerted on each atom, based on Born effective charge (Section S3, Supporting Information). Variations in Born effective charges among ions, along with induced interatomic interactions, enable the modulation of the polarization switching barrier under external electric fields. Consistent with DFT findings, the energy barrier for polarization reversal decreases under a negative electric field, leading to depolarization and destabilization of the FE domain. For instance, partial depolarization within the FE domain transitions to AFE coupling at E = −0.1 V Å^−1^. As the field strength increases, the proportion and area of AFE‐coupled domains expand, resulting in larger domains and domain fusion where two or more AFE domains merged into a larger AFE domain during their growth. At E = −0.8 V Å^−1^, a significantly enlarged central AFE domain emerges, disrupting the boundary between FE and AFE domains, effectively dividing the FE domain (**Figure** **4**a; Figures S14 and S15, Supporting Information). Conversely, under a positive electric field aligned with the polarization direction in the AA region, the FE domain remains stable. The Cu cations in the AFE domain shift toward the polarization direction, forming the polarized islands. Due to the higher potential barrier for AFE‐to‐FE transition compared to the depolarization process (Figure 1a), the AFE domain exhibits better stability under the same field intensity. Even at E = 0.8 V Å^−1^, the AFE domain remains relatively undisturbed. To quantitatively describe these findings, Figure 4a illustrates the percentage of structures in which polarization within the FE domain remains unchanged under an external electric field over 100 ps. The results show a nearly linear decrease (increase) in FE domain size in the AA region under negative (positive) electric fields, respectively.

The electric field not only influences domain size but also significantly impacts its lifetime, a key indicator of FE domain's stability. To quantify this effect, we calculated the autocorrelation of polarization in the AA‐stacked region, where the interlayer coupling is ferroelectric. As shown in Figure 4b, when the electric field intensity ranges from −0.1 to −0.5 V Å^−1^, the autocorrelation function is between 0.99 and 1, suggesting that the polarization state is stable over time. However, at −0.8 V Å^−1^, the autocorrelation function decreases significantly, reflecting transient polarization within the FE domain and the disruption of stable domain structures. In contrast, under a positive electric field, the autocorrelation function remains almost unchanged up to 0.8 V Å^−1^, indicating the enhanced stability of the FE domain in the AA‐stacked region (see Figures S14 and S15, Supporting Information).

In addition to electric fields, tensile strain along the <100> direction can also influence the polarization domain in the twisted structures. It is observed that the width of the FE domain is shrinking notably at 220 K (Figure 4c,d; Figures S16 and S17, Supporting Information), while the domain stability is weakened by external strain as evidenced by the decreasing percentage of the structures that can retain polarization. The impact of strain is twisting angle dependent, the structures with larger twist angles exhibit lower resilience to strain, a more rapid decay of polarized domains is found (Figure 4c). The polarization lifetime, however, is less pronounced than that of thermal fluctuations or electric fields. Although the autocorrelation decreases slightly with increasing strain, it remains close to 1, indicating relatively stable polarization over time (Figure 4d). This is because tensile strain induces only minor atomic displacements, unlike the significant atomic rearrangements caused by thermal fluctuations or electric fields. Consequently, the overall polarization within the FE domain is largely stabilized, despite a visible reduction in domain width.

## 3 Conclusion

In conclusion, a joint DFT and DeepMD simulations were employed to investigate polarization domain evolution in twisted CIPS bilayers. Our DFT calculations reveal that bilayer CIPS with different stacking configurations exhibit distinct polarization switching barriers due to stacking‐dependent interlayer interactions and electron transfer, providing the theoretical foundation for our findings. Using machine learning methods and the developed DP potential, large‐scale molecular dynamics simulations confirm the presence of FE domains in twisted CIPS bilayers, where the AA and AB regions of the Moiré structure exhibit interlayer FE and AFE coupling, respectively. The polarization domains and their stabilities are shown to be significantly influenced by thermal disturbances and externally applied electric fields, while demonstrating strong resistance to tensile strain. Our research offers a novel pathway for creating and manipulating polarization domains.

## 4 Experimental Section

### DFT Calculation for DL Potential Database and Switching Barrier

All DFT calculations were performed using the Vienna Ab Initio Simulation Package (VASP).^[^ ^41^, ^42^ ^]^ The exchange‐correlation functional was treated with the optB86 approximation,^[^ ^43^ ^]^ incorporating van der Waals corrections. The interaction between valence electrons and ion cores was described by the projected augmented wave (PAW) method and the Brillouin zone was sampled using the Monkhorst−Pack mesh with a reciprocal space resolution of 0.20 Å^−1^. The energy cutoff for the plane wave basis was set to 500 eV. All atoms were allowed to relax until the Hellmann‐Feynman forces were smaller than 0.001 eV Å^−1^, and the convergence criterion for the electronic self‐consistent loop was set to 10^−6^ eV. A vacuum region of 20 Å was included to eliminate the artifactual interactions between periodic images. To replicate the domain wall motion, the climbing image nudged elastic band (CI‐NEB) method was employed to determine the minimum energy path from the FE state to the AFE state.^[^ ^44^, ^45^ ^]^

### DL Potential Training and MD Simulation

The DeepMD‐kit^[^ ^33^ ^]^ was employed as the training package with the descriptor type set to “se_e2_a”. A cutoff value of 6.0 Å and a smooth radius of 2.0 Å were chosen to ensure descriptor smoothness. The embedding neural network used for descriptor training had a size of (25,50 100), while the fitting neural network had 240 neurons in each of its three layers. In the loss function setting, the weight *P_e_* was progressively increased from 0.02 to 2, while the weight *P_f_* was gradually reduced from 1000 to 1. Additionally, the virial weight *P_v_* was 0. The learning rate was initially set at 0.001 and decayed to 3.51e‐08 after 10 000 steps. The entire training process spanned 5 000 000 steps. The loss function^[^ ^35^ ^]^ in Deepmd‐kit, including the physic information of force and virial. Shows in the following equation:

(1) $$L\;\;\left(p_{e},p_{f},p_{v}\right)=\frac{p_{e}}{N}\Delta E^{2}+\frac{p_{f}}{3N}\sum_{i}{\left|\Delta F_{i}\right|}^{2}+\frac{p_{v}}{9N}|\left|\Delta v\right|{|}^{2}$$

where the three items Δ*E*, Δ*F*, and Δ*v* mean root mean square (RMS) error in energy, force, and virial. The three parameters *p_e_*,*p_f_* *and* *p_v_* is the weights of this three information.

The MD simulations were performed using the Large‐scale Atomic/Molecular Massively Parallel Simulator (LAMMPS).^[^ ^46^ ^]^ Non‐periodic boundaries were applied in the out‐of‐plane direction to simulate the bilayer structure, while periodic boundary conditions were used in the in‐plane directions to minimize edge effects. The simulations were conducted in the canonical ensemble (NVT), with temperature control maintained via the Nosé–Hoover thermostat.^[^ ^47^, ^48^ ^]^

## Conflict of Interest

The authors declare no conflict of interest.

## Author Contributions

D.B. and J.L. have made equal contributions to this work and can be recognized as co‐first authors. L.K. conceived the idea and supervised the research with Y.G. D.B. designed the MD simulation process and models. D.B. trained the DLMD potential and conducted the MD simulations, assisted by Y.N. J.L. executed the DFT simulations. D.B. analyzed the data with contributions from J.L., L.K., A.K., and D.Q. D.B. composed the manuscript with input from J.L. and L.K. All authors have approved the final version of the manuscript.

## Supporting information



Supporting Information

## Data Availability

The data that support the findings of this study are available from the corresponding author upon reasonable request.
