# Supplementary material for: Ferroelectric Domains and Evolution Dynamics in Twisted CuInP2S6 Bilayers
Source: Small Methods. 2025 Jun 20;10(1):2500683. doi: 10.1002/smtd.202500683 (PMC12790366; doi:10.1002/smtd.202500683)
Supplement: Supplementary file 1 — Supporting Information [file SMTD-10-2500683-s001.docx]

Supporting Information

**Ferroelectric Domains and Evolution Dynamics in Twisted CuInP_2_S_6_ Bilayers**

*Dongyu Bai^1₸^, Junxian Liu^1₸^, Yihan Nie^2^, Yuantong Gu^1^, Dongchen Qi^3^,* *Arkady Krasheninnikov^4^, Liangzhi Kou^1^**

1. School of Mechanical, Medical and Process Engineering, Queensland University of Technology, Brisbane, Queensland 4001, Australia
2. College of Civil Engineering and Architecture, Zhejiang University, Hangzhou 310058, China
3. School of Chemistry & Physics, Queensland University of Technology, Brisbane, Queensland 4001, Australia
4. Institute of Ion Beam Physics and Materials Research, Helmholtz-Zentrum Dresden-Rossendorf, 01328 Dresden, Germany

Corresponding author email: [*Liangzhi.kou@qut.edu.au*](mailto:Liangzhi.kou@qut.edu.au)

1. **Calculation Details**
   1. **The relative energy of different stacking configurations**

**
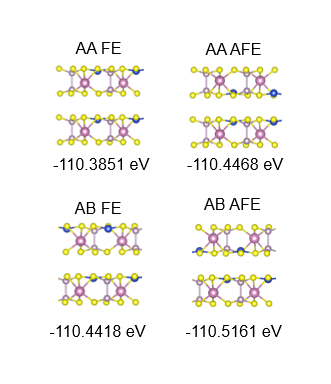
**

**Figure S1.** The relative energy of different stacking configurations.

- 1. **DFT calculation for different stacking state**

To accurately describe the interlayer interactions in different stacking configurations, the AB unit cell was used as the initial structure, with the top layer gradually sliding according to the vector $\boldsymbol{r}=i\boldsymbol{a}_{\boldsymbol{1}}+j\boldsymbol{a}_{\boldsymbol{2}}$, where $i$ , $j$ $\epsilon$ [0,1], $\boldsymbol{a}_{\boldsymbol{1}}$ and $\boldsymbol{a}_{\boldsymbol{2}}$ are lattice vectors of the unit cell. A total of 36 symmetric points were selected for structural relaxation, with $i$ = 6 and $j$ = 6, to simulate the local lattice mismatch with in the moire superlattice. To prevent lateral displacement of the top layer atoms during the relaxation process, the in-plane movement of all atoms in the bilayer CIPS was fixed, allowing relaxation only in the out-of-plane direction.


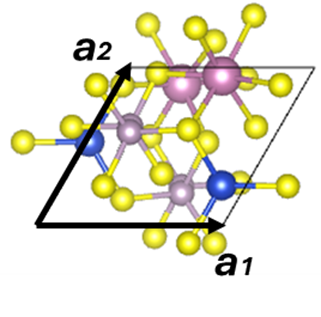


**Figure S2.** Different stacking configurations generated by laterally sliding the top layer.

- 1. **Time autocorrelation function**

The time autocorrelation function of polarization $\vec{p_{i}}$ was calculated as:

| $\alpha\left( t \right)=\frac{<\int\vec{p_{i}}(t_{0})\vec{p_{i}}\left( t_{0}+t \right)dt_{0}>}{\alpha_{i}(0)}$ | (S1) |
| --- | --- |

to describe the difference of the polarization at the time interval of $t$. $\vec{D_{i}}\left( t_{0} \right)$ is the value of the dipole moment of chunk $i$ at $t_{0}$. $t$ is the time interval. The autocorrelation function starts at $t$ =0, $\alpha_{i}(0)$ normalize the starting point to 1 for comparison. If the autocorrelation function remains close to 1 over a given period, the polarization is stable during that time.

1. **Training and validation details of Deep Learning potential**

Using “dp-test” command in DeepMD-kit package ^[1, 2]^ to test the precision of Deep Learning potential within the training domain. The number of test frames is set to 30 and test results are shown in Figure S2, 3.


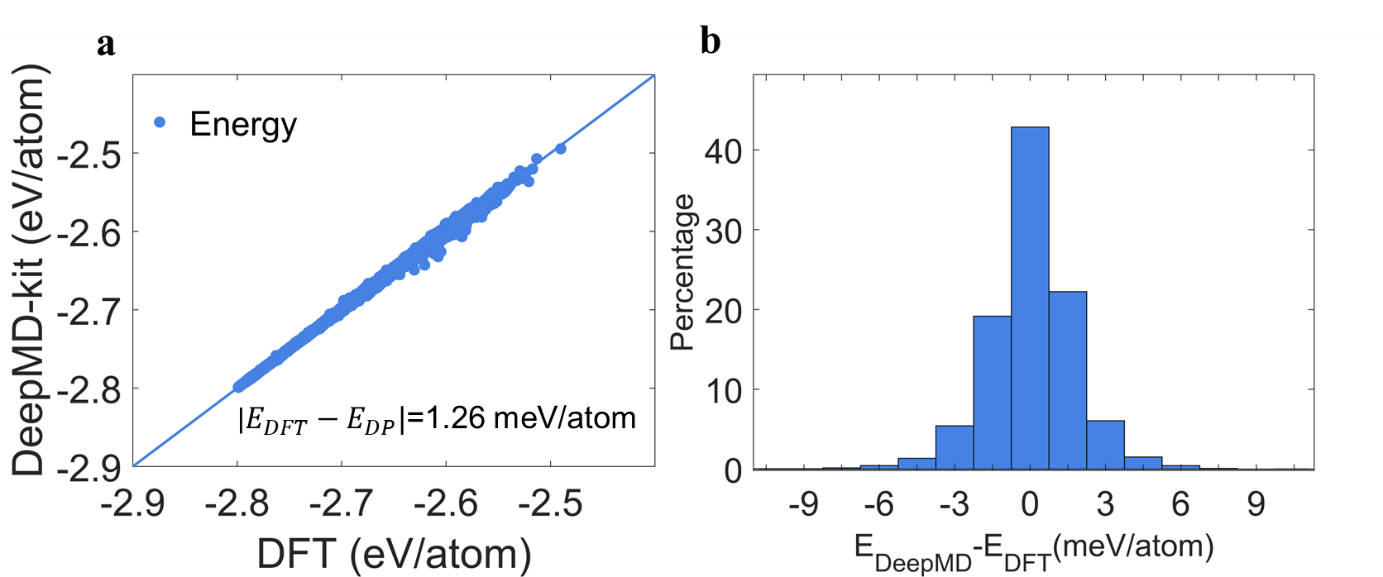


**Figure S3** a) Comparison of DFT and DLMD energy per atom. b) The distribution of absolute error between DFT and DLMD energy


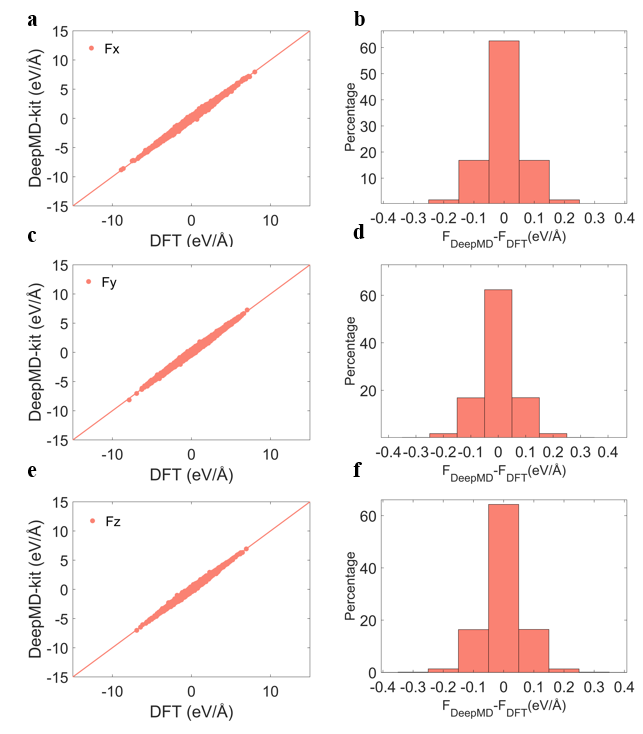


**Figure S4** a,c,e) Comparison of DFT and DPMD atomic forces for configurations in the training database. b,d,f) The distribution of absolute error between DFT and DLMD atomic force.


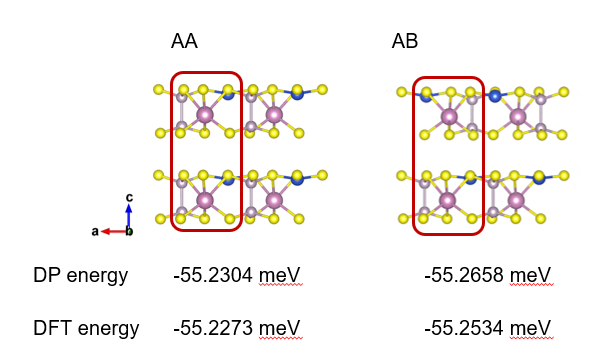


**Figure S5** DP-predicted and DFT-calculated primitive unit cell energies for the metastable AA and ground-state AB stacking configurations.

1. **The calculation of Born effective charge**

In this work, the polarization and the applied electric field on the unit CIPS are calculated using the Born effective charge tensor. The polarization $P$ of CIPS unit was calculated by the following formula:

| $P=\frac{1}{V}\sum_{i} \boldsymbol{Z}_{\boldsymbol{i}}^{\boldsymbol{*}}\boldsymbol{u}_{\boldsymbol{i}}$ | (S2) |
| --- | --- |

Where $V$ is the volume of the CIPS unit, $\boldsymbol{Z}_{\boldsymbol{i}}^{\boldsymbol{*}}$ is the Born effective charge tensor. $\boldsymbol{u}_{\boldsymbol{i}}$ refers to the atomic coordinate displacement relative to the non-polar state structure of CIPS. All polarizations in this work are considered solely in the out-of-plane direction. Therefore, when calculating the Bonn effective charge tensor $\boldsymbol{Z}_{\boldsymbol{i}}^{\boldsymbol{*}}$, only the out-of-plane components are taken into account. Using density-functional perturbation theory with symmetry constraints, the Bonn effective charge is calculated in VASP ^[3]^. Since P₂S₆ can be treated as a rigid ion ^[4]^, the calculated Bonn effective charges are $\boldsymbol{Z}_{\boldsymbol{Cu}}^{\boldsymbol{*}}$ = 0.61, $\boldsymbol{Z}_{\boldsymbol{In}}^{\boldsymbol{*}}$ = 2.8 and $\boldsymbol{Z}_{\boldsymbol{P}\boldsymbol{2}\boldsymbol{S}\boldsymbol{6}}^{\boldsymbol{*}}$ = 3.4.

In MD simulations, the influence of an external out-of-plane electric field on the system is modelled by applying an electric field force $F$ to each atom. The field force $F$ was calculated by

| $F=EZ_{i}^{*}$ | (S3) |
| --- | --- |

Where $E$ is the Electric field intensity.

1. **Twisted models**

**
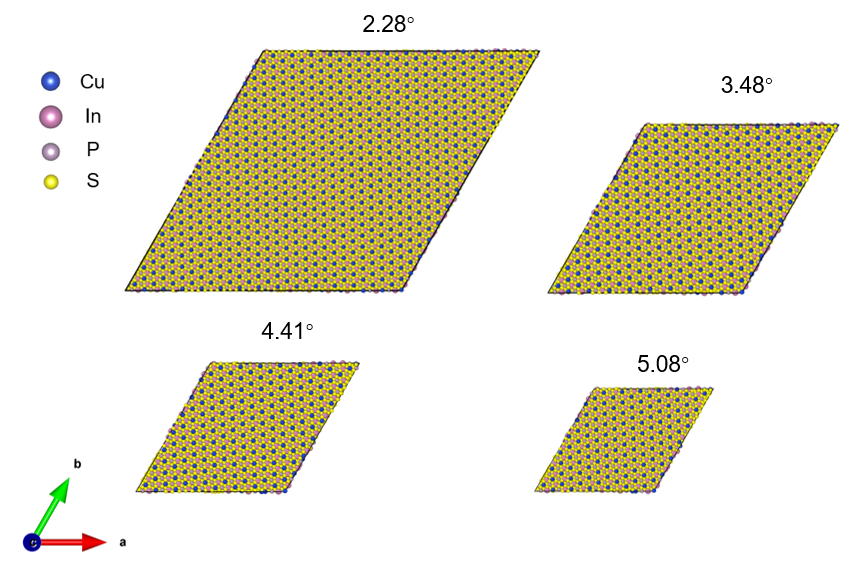
**

**Figure S6** Diagrams of four models with twist angles of 2.28°, 3.48°, 4.41°, and 5.08°.


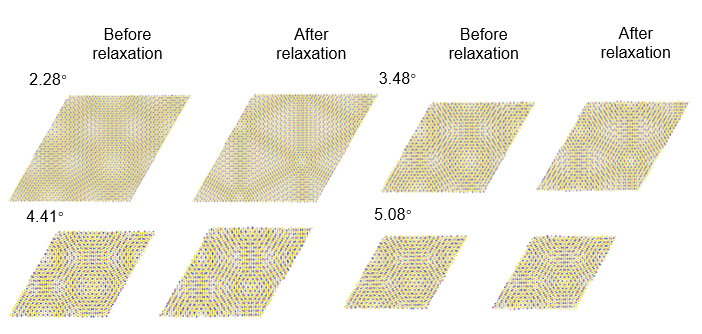


**Figure S7** Initial Moiré structures (left) constructed with four different twist angles (2.28°, 3.48°, 4.41°, and 5.08°), showing equal areas of AA and AB stacking domains. Fully relaxed configurations (right) using the Deep Potential (DP) model, the AB domains expand while the AA domains shrink.

**
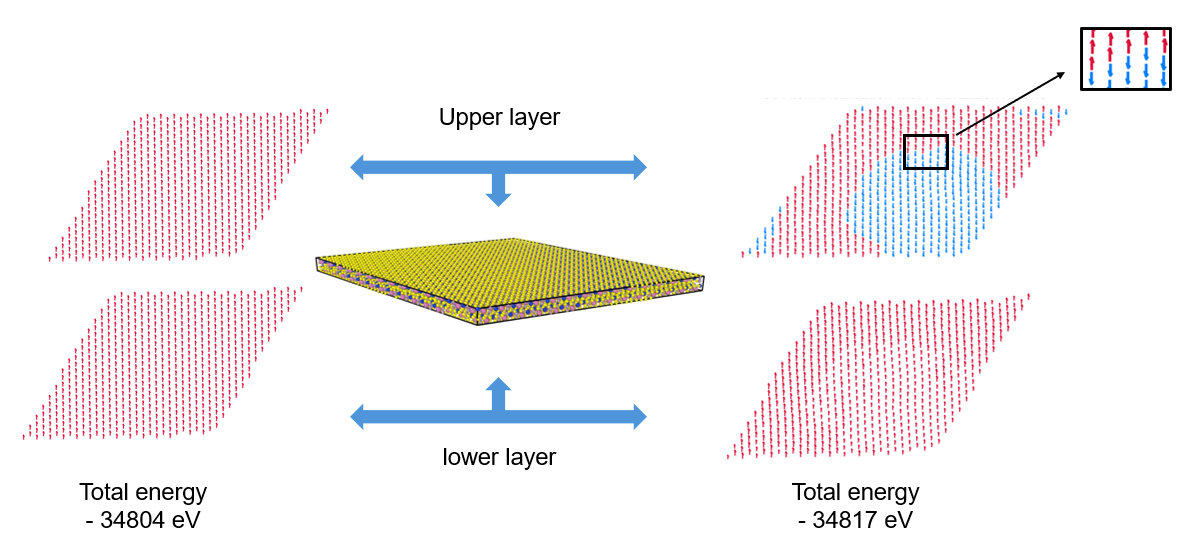
**

**Figure S8** The energy of ferroelectric coupling configurations and stacking-dependent polar domain coupling (antiferroelectric coupling in the AA-stacked region and ferroelectric coupling in the AB-stacked region).

1. **Twist-induced polarization domain distribution and thermal stability**

The following is the heatmaps of the polarization distribution at 2.28°, 3.48°, 4.41° and 5.08° four twisted angles for temperatures ranging from 220K to 320K. As the twist angle increases, the AFE domain structure decreases. Although both AFE and FE domains in all angular models are disrupted with rising temperatures, the domains in smaller-angle models remain relatively intact at higher temperatures. This demonstrates that smaller twist angles enhance the thermal stability of the polarization domain.

**
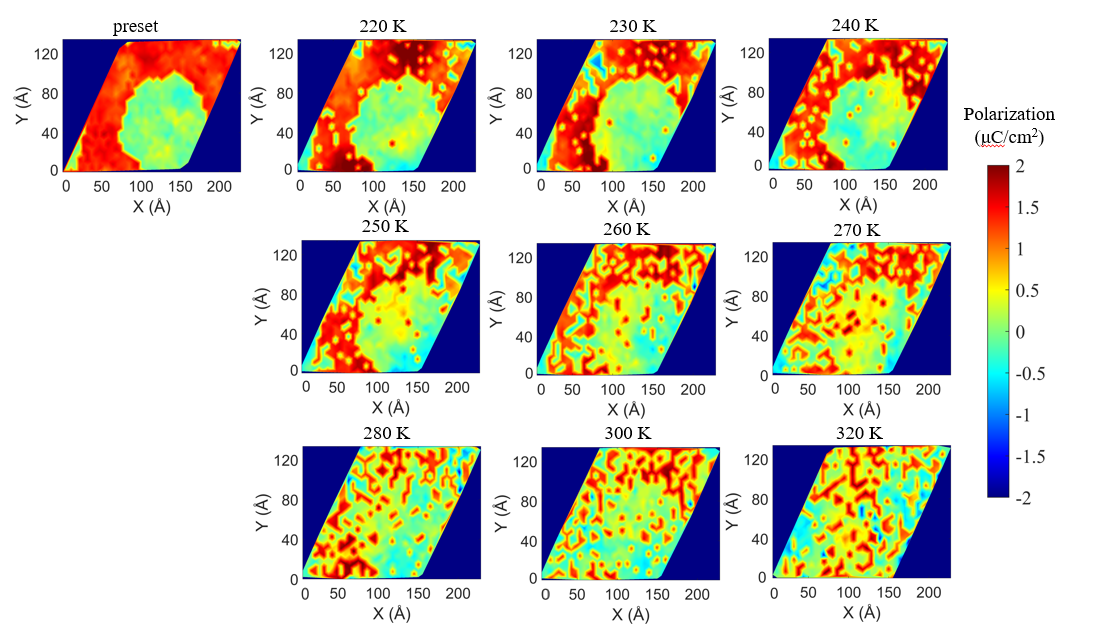
**

**Figure S9** Heatmap of polarization distribution at various temperatures when the twist angle bilayer CIPS is 2.28°. Initially, the AA structure is situated at the center of the AFE domain, while the AB structure predominantly constitutes the FE domain. During the MD relaxation process, the dominant structure in FE transited from AB to AA.

**
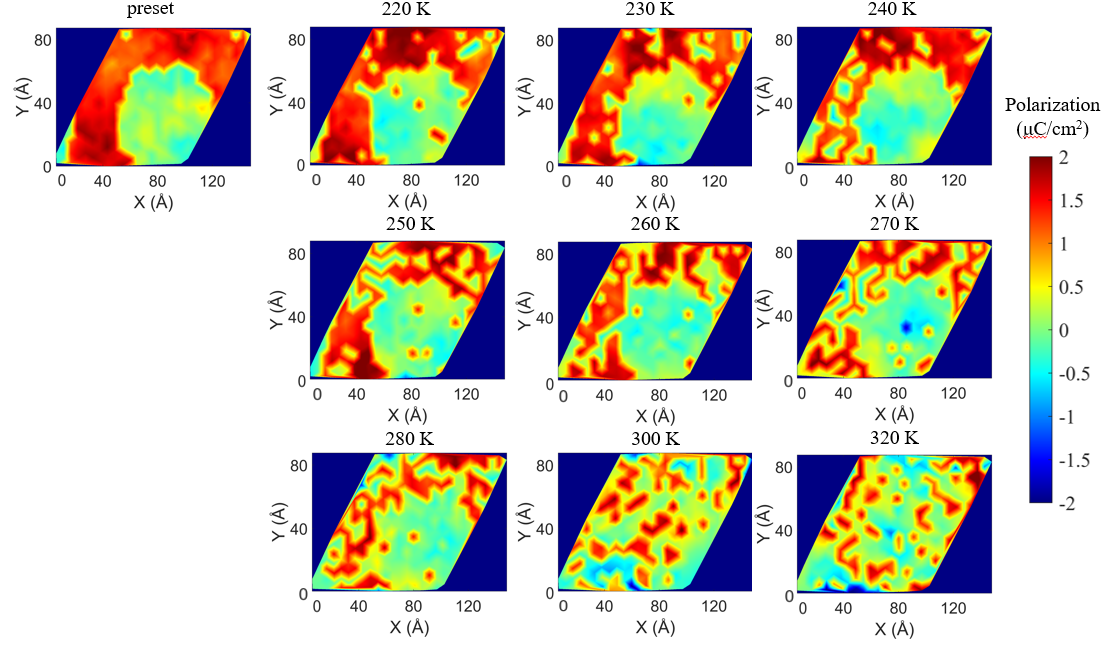
**

**Figure S10** Heatmap of polarization distribution at various temperatures when the twist angle bilayer CIPS is 3.48°.

**
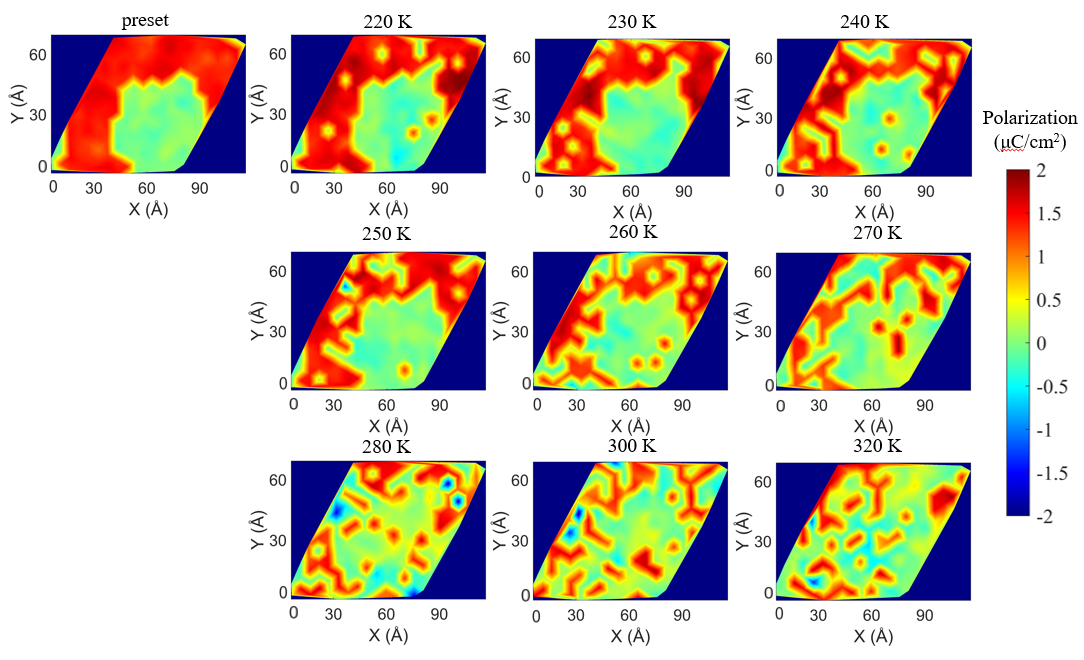
**

**Figure S11** Heatmap of polarization distribution at various temperatures when the twist angle bilayer CIPS is 4.41°.

**
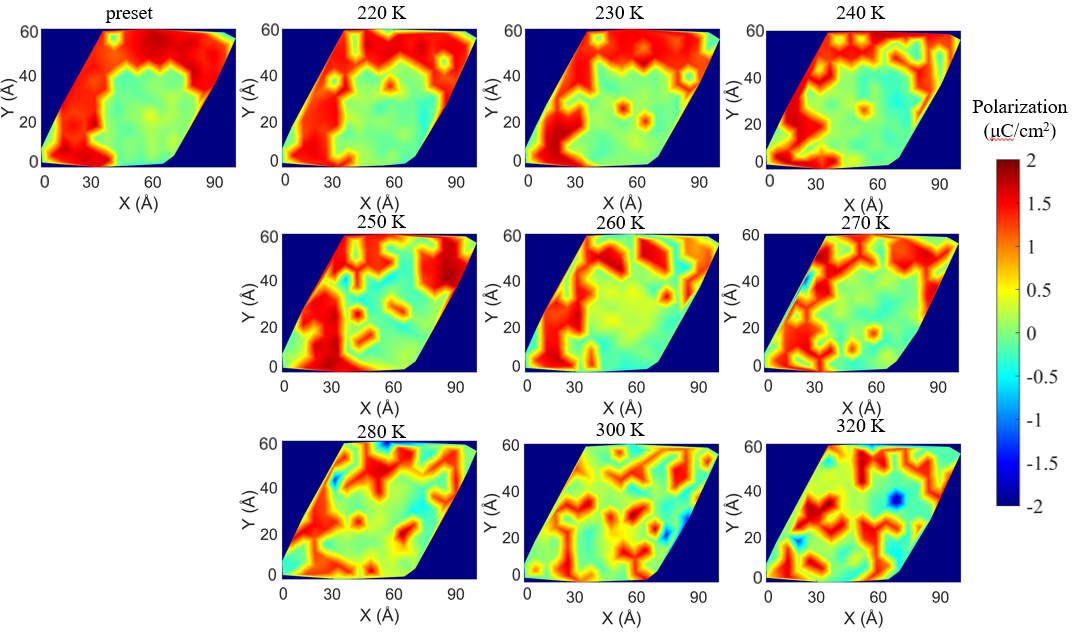
**

**Figure S12** Heatmap of polarization distribution at various temperatures when the twist angle bilayer CIPS is 5.08°.

1. **The first switching barrier of Cu Dipole with external electric field**
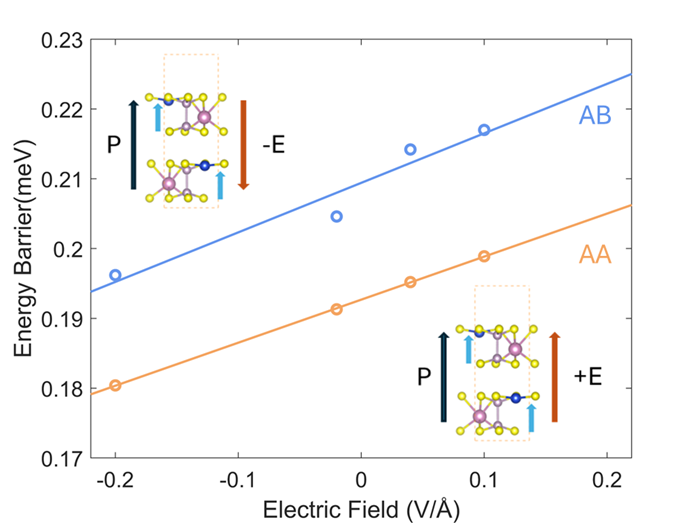


**Figure S13** Variation of the first energy barrier with an external electric field in DFT calculation.

1. **Polarization domain distribution under external electric field**

The following figure shows the polarization distribution under an applied electric field ranging from ±0.1V/Å to ±0.9 V/Å at two twist angles, 2.28° and 4.41°, at a temperature of 220 K. When the electric field is applied in the inward direction, aligning with the Cu dipole movement, the energy barrier for switching is reduced, causing the red polarization domain to become unstable and shrink in size. Once the electric field strength exceeds +0.8 V/Å, the energy barrier decreases to a point where the polarization domain undergoes significant depolarization, compromising its structural integrity. Conversely, when the electric field is reversed, the energy barrier from the FE to AFE state increases, stabilizing the polarization domain. In the non-polarized structure, as the reversed electric field strength continues to increase, part of the structure reverts to the polarized state, causing the non-polarized region to shrink. This effect is even more pronounced in structures with larger twisted angles.

**
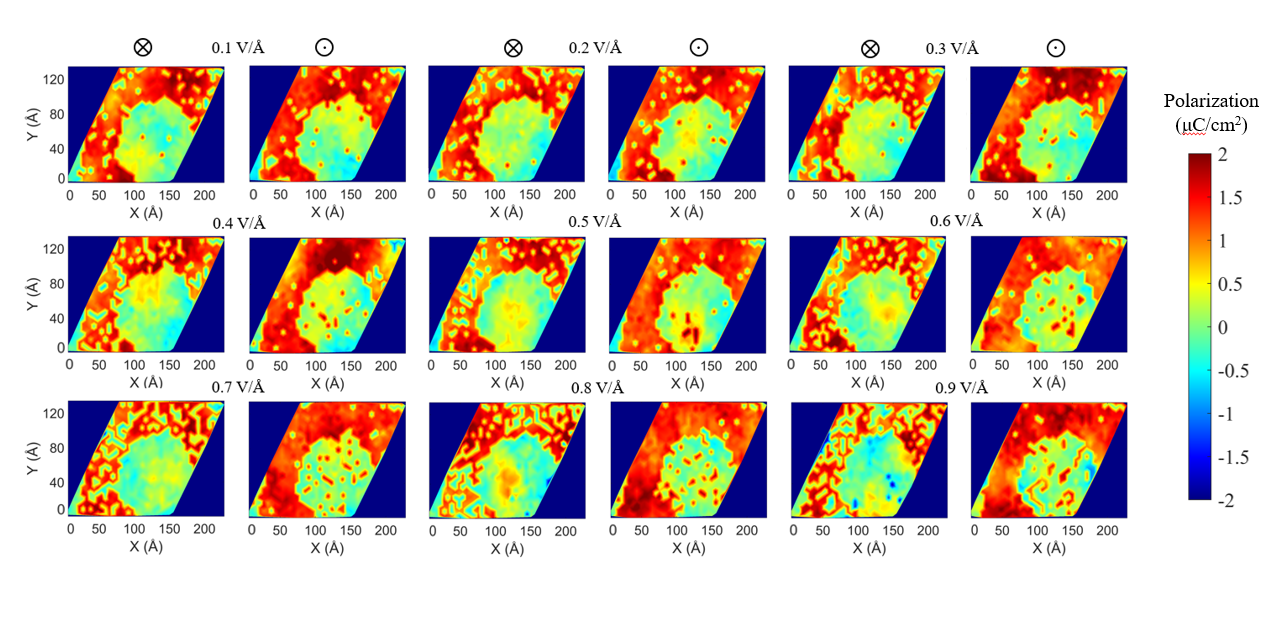
**

**Figure S14** Heatmap of polarization distribution at external vertical electrical field when the twist angle bilayer CIPS is 2.28°.

**
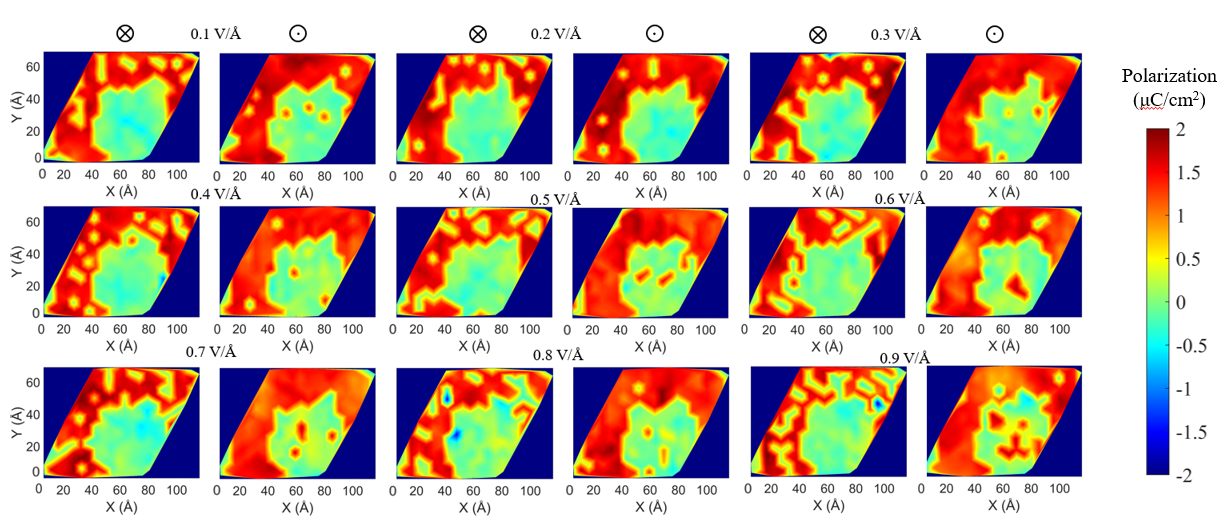
**

**Figure S15** Heatmap of polarization distribution at external vertical electrical field when the twist angle bilayer CIPS is 4.41°.

1. **Polarization domain distribution influenced by uniaxial in-plane strain**

A tensile strain ranging from 0.3% to 2.3% was applied to structures with torsion angles of 2.28° and 4.41° at a temperature of 220 K. The tensile strain facilitated polarization inversion and significantly reduced the area of the FE domain. Under higher strain, the structural response is more pronounced for smaller angles.

**
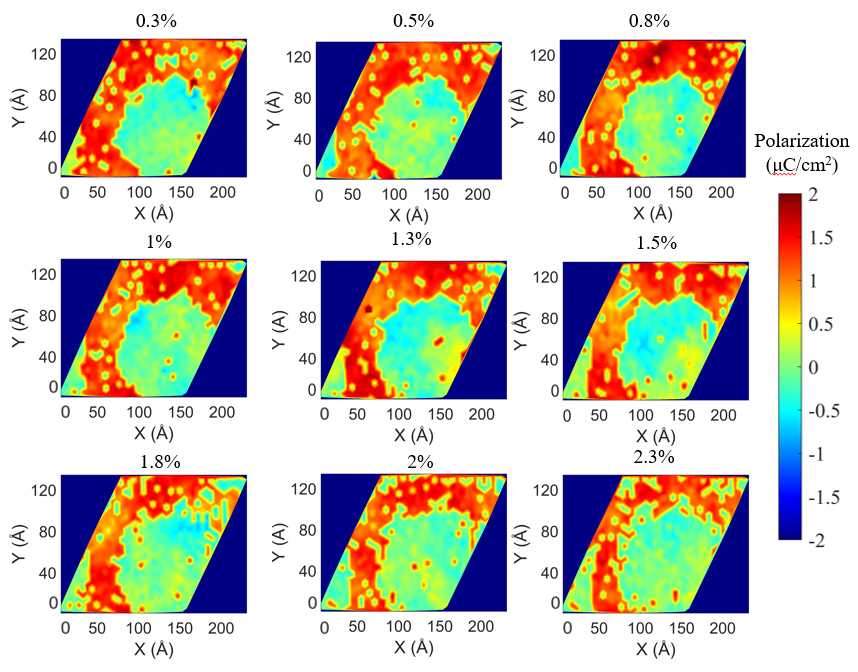
**

**Figure S16** Heatmap of polarization distribution under uniaxial in-plane strain when the twist angle bilayer CIPS is 2.28°.

**
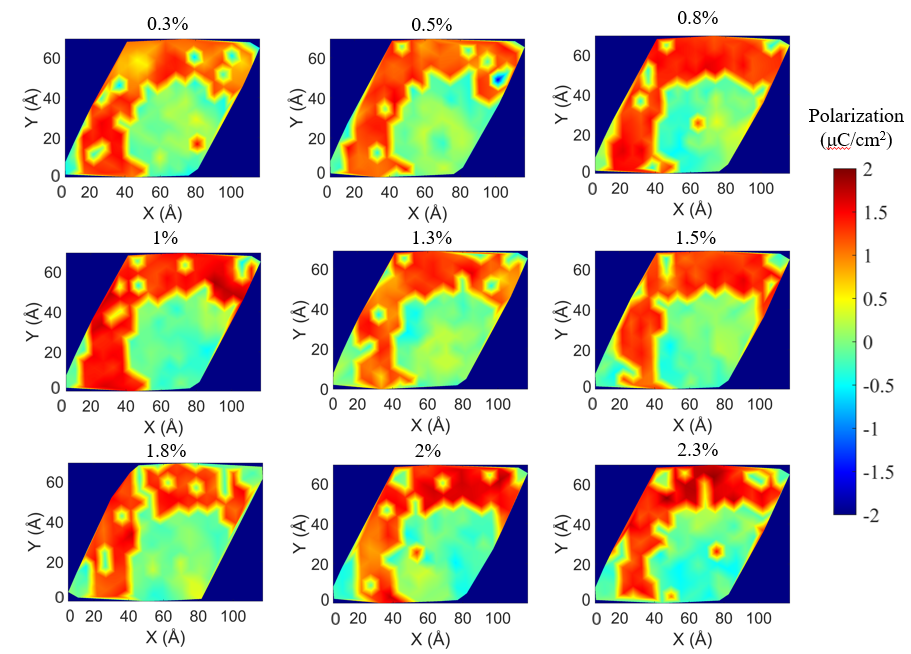
**

**Figure S17** Heatmap of polarization distribution under uniaxial in-plane strain when the twist angle bilayer CIPS is 4.41°.

1. **Polarization distribution function**

The polarization distribution function represents the distribution of polarization at varying radial distances, centered on the geometric center $\vec{r_{o}}$ of the initial AFE domain in each structure, $\vec{r_{o}}=\frac{1}{n} \sum_{i=1}^{n} (\boldsymbol{x}_{\boldsymbol{i}},\boldsymbol{y}_{\boldsymbol{i}},\boldsymbol{z}_{\boldsymbol{i}}).$ Here, $(\boldsymbol{x}_{\boldsymbol{i}},\boldsymbol{y}_{\boldsymbol{i}},\boldsymbol{z}_{\boldsymbol{i}})$ is the position vector of the $i$-th cell, and structure $\vec{r_{o}}$ represents the position vector of the geometric center. The polarization distribution function quantifies and visually compares the effects of twist angle, temperature, and external field on the size of the polarization domain.

The PDF was calculated as follows:

| $P(r)=\int_{rmin}^{rmax} P\left( r^{'} \right)dr^{'}$ | (S4) |
| --- | --- |

$r$ is the distance from calculation point to the geometric center $\vec{r_{o}}$.

.
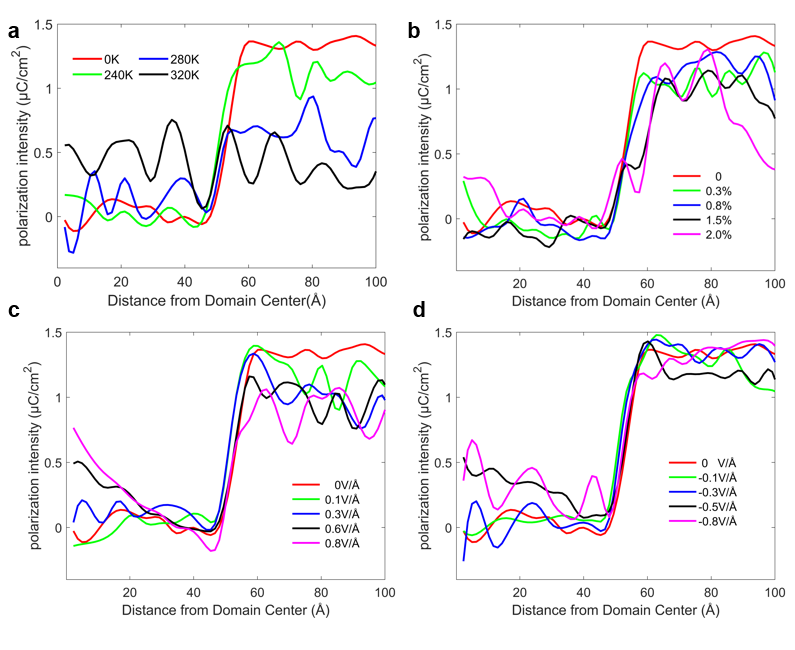


**Figure S18** Polarization distribution function (PDF) for the twist angle of 2.28 ⁰ bilayer CIPS. a) PDF at different temperatures. b) PDF under different strain. c) PDF under different inward electric field. d) PDF under different outward electric field.

**Reference**

[1] H. Wang, L. Zhang, J. Han, W. E, Comput Phys Commun 2018, 228, 178-184.

[2] L. Zhang, J. Han, H. Wang, R. Car, W. E, Phys. Rev. Lett. 2018, 120, 143001.

[3] P. Ghosez, J.-P. Michenaud, X. Gonze, Phys. Rev. B 1998, 58, 6224.

[4] R. He, H. Wang, F. Liu, S. Liu, H. Liu, Z. Zhong, Phys. Rev. B 2023, 108, 024305.
